# A single nucleotide polymorphism in *CAPN1 *associated with marbling score in Korean cattle

**DOI:** 10.1186/1471-2156-9-33

**Published:** 2008-04-19

**Authors:** Hyun Sub Cheong, Du-Hak Yoon, Byung Lae Park, Lyoung Hyo Kim, Joon Seol Bae, Sohg Namgoong, Hae Won Lee, Chang Soo Han, Ji On Kim, Il-Cheong Cheong, Hyoung Doo Shin

**Affiliations:** 1 Department of Genetic Epidemiology, SNP Genetics, Inc., Rm 1407, Complex B, WooLim Lion's Valley, 371-28, Gasan-Dong, Geumcheon-Gu, Seoul, 153-803, Korea; 2 Du-Hak Yoon, Il-Cheong Cheong. National Livestock Research Institute, RDA, Suwon, 441-706, Korea; 3 Department of Life Science, Sogang University, 1 Shinsu-dong, Mapo-gu, Seoul, 121-742, Korea

## Abstract

**Background:**

Marbling score (MS) is the major quantitative trait that affects carcass quality in beef cattle. In this study, we examined the association between genetic polymorphisms of the micromolar calcium-activated neutral protease gene (micro-calpain, *CAPN1*) and carcass traits in Korean cattle (also known as Hanwoo).

**Results:**

By direct DNA sequencing in 24 unrelated Korean cattle, we identified 39 sequence variants within exons and their flanking regions in *CAPN1*. Among them, 12 common polymorphic sites were selected for genotyping in the beef cattle (*n *= 421). Statistical analysis revealed that a polymorphism in the 3'UTR (c.2151*479C>T) showed significant association with MS (*P*^*cor*. ^= 0.02).

**Conclusion:**

Our findings suggest that polymorphisms in *CAPN1 *might be one of the important genetic factors involved in carcass quality in beef cattle, although it could be false positive association.

## Background

Genetic improvement has long been considered an important factor in the competitiveness of beef cattle production. Identification of the genes and/or polymorphisms underlying quantitative/qualitative traits, and an understanding of how these genes/polymorphisms interact with the environment or with other genes affecting economic traits might be the keys to successful application of marker-assisted selection in the commercial animal population. As one of these economic traits, marbling is intramuscular fat that gives meat flavor and tenderness. Thus, an increase in the degree of marbling raises the level of meat quality.

Calpain is a ubiquitous cytoplasmic cysteine protease, the activity of which is absolutely dependent on calcium [1]. Two genes of calpain (CAPN1 [macro-calpain] and CAPN2 [mili-calpain]) have been identified [2]. CAPN1 degrades myofibrillar proteins under postmortem conditions and appears to be the primary enzyme in the postmortem tenderization process [3-6]. Regulation of CAPN1 activity has been correlated with variation in meat tenderness, and previous studies also identified a quantitative trait locus influencing meat tenderness on chromosome 29 where CAPN1 lies [7-9].

In the CAPN1 gene, more than 100 single nucleotide polymorphisms (SNPs) have been identified in *Bos indicus *or *Bos taurus *[8,10,11] (S. N. White and T. Smith, unpublished data). Among them, four polymorphisms, two non-synonymous SNPs (G316A and V530I), and two intronic SNPs (C4685T and C4751T) have been found to have significant effects on meat tenderness [10,12-16].

Association studies of SNPs in *CAPN1 *with carcass traits have been performed, but there is no publicly available evaluation of the association of these SNPs in Korean cattle (*Bos taurus coreanae*, also known as Hanwoo). Thus, the objective of this study was to discover polymorphisms and assess the association, including that of reported SNPs, in Korean cattle.

## Results and Discussion

**Figure 1:**
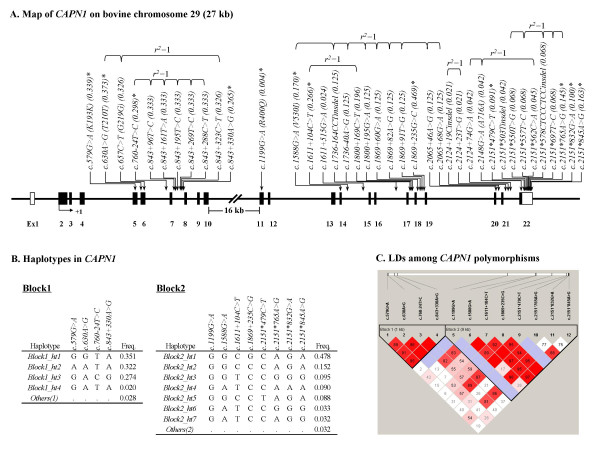
**Gene map, haplotypes, and linkage disequilibrium in *CAPN1***. **A**. Gene map and polymorphisms in *CAPN1 *on chromosome 29. The coding exon is marked by black blocks, and 5' and 3' UTRs by white blocks. The first base of the translational site is denoted as nucleotide +1. Asterisks (*) indicate polymorphisms genotyped in a larger Korean native cattle cohort (n = 421). **B**. Haplotypes in *CAPN1*. Haplotypes with frequency >0.02 are presented. *Others *contain rare haplotypes: (1)*AGTA, GACA*, and *GGCA*; and (2) *GATCCGGA, GATGCAGA, GGCGCAAA*, and *GATGCGGA*. **C**. Linkage disequilibrium (LD) and LD blocks among *CAPN1 *polymorphisms are shown using Haploview. The color code on the Haploview plot follows the standard color scheme: white (|D'| < 1, LOD < 2); shades of pink/red (|D'| < 1, LOD ≥ 2); blue (|D'| = 1, LOD < 2); bright red (|D'| = 1, LOD ≥ 2). The numbers in cells are D' values. However, the D' values of 1.0 are not shown (empty).

By direct DNA sequencing, 39 polymorphisms were identified within exons and their flanking regions of *CAPN1*: 6 in coding exons, 10 in 3'UTR, and 23 in introns. The locations and allele frequencies of the polymorphisms are shown in Table 1 and Figure 1A. Among those identified, 12 polymorphisms (c.579G>A [K193K], c.630A>G [T210T], c.760-24T>C, c.843+330A>G, c.1199G>A [R400Q], c.1588G>A [V530I], c.1611+104C>T, c.1869+235C>G, c.2151*479C>T, c.2151*765A>G, c.2151*832G>A, and c.2151*845A>G) were selected for larger-scale genotyping based on location (polymorphisms in exons were preferred), minor allele frequency exceeding 0.05, LD (a polymorphism was chosen if it was in absolute LD [*r*^2 ^= 1] with one or more other polymorphisms), and haplotype tagging status (Table 2 and Figure 1A). The minor allele frequencies of genotyped polymorphisms in Korean cattle (n = 421) are described in Table 1. Pairwise linkage disequilibrium analysis with the 12 polymorphisms showed that CAPN1 can be parsed into two LD blocks (*Block1 *and *Block2*), the 1.5 kb region spanning from exon5 to intron7 (*Block1*) and the 9 kb region from exon11 to exon22 (*Block2*). The distance between two adjacent SNPs (*c.843+330A>G *in *Block1 *and *c.1199G>A *in *Block2*) is approximately 13 kb (Figures 1A and 1C). There were three and five common haplotypes (freq. > 0.05) in *Block1 *and *Block2*, respectively (Figures 1B and 1C).

**Table 1:** Genotype and allele frequencies of 39 polymorphisms in *CAPN1*

SNP	Position	AA change	Genotypes and number of animal			Minor allele frequency	Heterozygosity	HWE**
c.579G>A*	Exon5	K193K	G (187)	AG (177)	A (53)	0.339	0.448	0.276
c.630A>G*	Exon6	T210T	A (174)	AG (180)	G (67)	0.373	0.468	0.078
*c*.657*C*>*T*	Exon6	G219G	C (13)	CT (5)	T (5)	0.326	0.44	0.015
c.760-24T>C*	Intron6	.	T (210)	CT (161)	C (43)	0.298	0.419	0.148
*c.843+96T>C*	Intron7	.	T (10)	CT (12)	C (2)	0.333	0.444	0.54
*c.843+161T>A*	Intron7	.	T (10)	AT (12)	A (2)	0.333	0.444	0.54
*c.843+195T>C*	Intron7	.	T (10)	CT (12)	C (2)	0.333	0.444	0.54
*c.843+269T>C*	Intron7	.	T (10)	CT (12)	C (2)	0.333	0.444	0.54
*c.843+288C>T*	Intron7	.	C (10)	CT (12)	T (2)	0.333	0.444	0.54
*c.843+323C>T*	Intron7	.	C (13)	CT (5)	T (5)	0.326	0.44	0.015
c.843+330A>G*	Intron7	.	A (224)	AG (156)	G (31)	0.265	0.39	0.596
c.1199G>A*	Exon11	R400Q	G (417)	AG (3)	A (0)	0.004	0.007	0.941
c.1588G>A*	Exon14	V530I	G (289)	AG (111)	A (15)	0.17	0.282	0.293
c.1611+104C>T*	Intron14	.	C (227)	CT (155)	T (33)	0.266	0.391	0.369
*c.1611+515G>A*	Intron14	.	G (20)	AG (1)	A (0)	0.024	0.046	0.911
*c.1736-164CCTinsdel*	Intron16	.	del (19)	insdel (4)	ins (1)	0.125	0.219	0.243
*c.1736-40A>G*	Intron17	.	A (19)	AG (4)	G (1)	0.125	0.219	0.243
*c.1800+169C>T*	Intron17	.	C (16)	CT (5)	T (2)	0.196	0.315	0.138
*c.1800+195G>A*	Intron17	.	G (19)	AG (4)	A (1)	0.125	0.219	0.243
*c.1869+60G>A*	Intron18	.	G (19)	AG (4)	A (1)	0.125	0.219	0.243
*c.1869+82A>G*	Intron18	.	A (19)	AG (4)	G (1)	0.125	0.219	0.243
*c.1869+91T>G*	Intron18	.	T (19)	GT (4)	G (1)	0.125	0.219	0.243
c.1869+235G>C*	Intron18	.	C (128)	CG (189)	G (102)	0.469	0.498	0.053
*c.2065+46A>G*	Intron20	.	A (19)	AG (4)	G (1)	0.125	0.219	0.243
*c.2065+68G>A*	Intron20	.	G (19)	AG (4)	A (1)	0.125	0.219	0.243
*c.2124+19Cinsdel*	Intron21	.	ins (23)	insdel (1)	del (0)	0.021	0.041	0.917
*c.2124+23T>G*	Intron21	.	T (23)	GT (1)	G (0)	0.021	0.041	0.917
*c.2124+74G>A*	Intron21	.	G (22)	AG (2)	A (0)	0.042	0.08	0.831
*c.2148G>A*	Exon22	A716A	G (22)	AG (2)	A (0)	0.042	0.08	0.831
c.2151*479C>T*	3'UTR	.	C (342)	CT (65)	T (5)	0.091	0.165	0.345
c.2151*503Tinsdel	3'UTR	.	ins (22)	insdel (2)	del (0)	0.042	0.08	0.831
c.2151*550T>G	3'UTR	.	T (19)	GT (3)	G (0)	0.068	0.127	0.731
c.2151*557T>C	3'UTR	.	T (19)	CT (3)	C (0)	0.068	0.127	0.731
c.2151*562C>A	3'UTR	.	C (20)	AC (2)	A (0)	0.045	0.087	0.823
c.2151*578CTCCCTCCinsdel	3'UTR	.	ins (19)	insdel (3)	del (0)	0.068	0.127	0.731
c.2151*697T>C	3'UTR	.	T (19)	CT (3)	C (0)	0.068	0.127	0.731
c.2151*765A>G*	3'UTR	.	A (307)	AG (96)	G (12)	0.145	0.247	0.187
c.2151*832G>A*	3'UTR	.	G (335)	AG (75)	A (4)	0.1	0.18	0.931
c.2151*845A>G*	3'UTR	.	A (293)	AG (112)	G (12)	0.163	0.273	0.744

*Frequencies of the six polymorphisms marked by asterisks were determined by genotyping 421 Korean native cattle. Frequencies of the remaining polymorphisms were determined by re-sequencing 24 unrelated Korean native cattle DNAs.

** *P-*values of deviation from Hardy-Weinberg equilibrium

**Table 2:** Association analyses of *CAPN1 *polymorphisms with carcass traits (CW and MS) among Korean native cattle

				Genotype				
						
Trait	Polymorphism	Position	Amino acid change	C/C	C/R	R/R	*P*	*P*^*cor*.^
						
				N(LSMEAN ± SE)	N(LSMEAN ± SE)	N(LSMEAN ± SE)		
CW	*c*.579*G*>*A*	Exon5	K193K	187(309.56 ± 2.58)	177(311.93 ± 2.57)	53(314.91 ± 4.99)	0.33	NS
	*c*.630*A*>*G*	Exon6	T210T	174(312.17 ± 2.61)	180(311.14 ± 2.53)	67(310.88 ± 4.20)	0.16	NS
	*c.760-24T>C*	Intron6	.	210(312.93 ± 2.24)	161(306.26 ± 2.72)	43(319.27 ± 4.92)	0.11	NS
	*c.843+330A>G*	Intron7	.	224(312.42 ± 2.21)	156(306.68 ± 2.65)	31(323.82 ± 6.18)	0.69	NS
	*c.1199G>A*	Exon11	R400Q	417(311.43 ± 1.50)	3(294.47 ± 19.76)	.	0.46	NS
	*c.1588G>A*	Exon14	V530I	289(311.78 ± 1.93)	111(309.62 ± 3.27)	15(311.41 ± 9.12)	0.84	NS
	*c.1611+104C>T*	Intron14	.	227(311.32 ± 2.26)	155(311.06 ± 2.74)	33(313.20 ± 5.98)	0.88	NS
	*c.1869+235C>G*	Intron18	.	128(313.33 ± 3.15)	189(308.92 ± 2.50)	102(312.55 ± 3.23)	0.73	NS
	c.2151*479C>T	3'UTR	.	342(311.37 ± 1.78)	65(304.72 ± 4.80)	5(321.32 ± 16.27)	0.49	NS
	c.2151*765A>G	3'UTR	.	307(310.08 ± 2.01)	96(310.63 ± 4.15)	12(311.52 ± 11.10)	0.23	NS
	c.2151*832G>A	3'UTR	.	335(311.67 ± 1.75)	75(309.30 ± 4.14)	4(315.85 ± 19.01)	0.84	NS
	c.2151*845A>G	3'UTR	.	293(308.97 ± 2.07)	112(312.82 ± 3.75)	12(317.88 ± 10.33)	0.11	NS
	*Block2_ht2*	.	.	300(310.77 ± 1.86)	95(314.78 ± 3.50)	15(300.73 ± 8.72)	0.85	NS
	*Block2_ht3*	.	.	335(310.25 ± 1.80)	72(316.69 ± 4.68)	3(303.60 ± 20.41)	0.46	NS

MS	*c*.579*G*>*A*	Exon5	K193K	187(2.10 ± 0.10)	177(2.39 ± 0.10)	53(2.00 ± 0.19)	0.85	NS
	*c*.630*A*>*G*	Exon6	T210T	174(2.19 ± 0.10)	180(2.29 ± 0.10)	67(2.09 ± 0.17)	0.64	NS
	*c.760-24T>C*	Intron6	.	210(2.18 ± 0.09)	161(2.26 ± 0.11)	43(2.16 ± 0.20)	0.69	NS
	*c.843+330A>G*	Intron7	.	224(2.18 ± 0.09)	156(2.21 ± 0.11)	31(2.16 ± 0.25)	0.78	NS
	*c.1199G>A*	Exon11	R400Q	417(2.21 ± 0.06)	3(1.77 ± 0.79)	.	0.47	NS
	*c.1588G>A*	Exon14	V530I	289(2.21 ± 0.08)	111(2.24 ± 0.13)	15(2.12 ± 0.37)	0.60	NS
	*c.1611+104C>T*	Intron14	.	227(2.19 ± 0.09)	155(2.17 ± 0.11)	33(2.56 ± 0.24)	0.22	NS
	*c.1869+235C>G*	Intron18	.	128(2.30 ± 0.13)	189(2.23 ± 0.10)	102(2.12 ± 0.13)	0.09	NS
	c.2151*479C>T	3'UTR	.	342(2.34 ± 0.07)	65(1.56 ± 0.19)	5(0.94 ± 0.65)	**0.0007**	**0.02**
	c.2151*765A>G	3'UTR	.	307(2.19 ± 0.08)	96(2.33 ± 0.17)	12(2.67 ± 0.45)	0.08	NS
	c.2151*832G>A	3'UTR	.	335(2.20 ± 0.07)	75(2.31 ± 0.17)	4(1.10 ± 0.76)	0.13	NS
	c.2151*845A>G	3'UTR	.	293(2.15 ± 0.08)	112(2.42 ± 0.15)	12(2.77 ± 0.41)	0.06	NS
	*Block2_ht2*	.	.	300(2.11 ± 0.07)	95(2.56 ± 0.14)	15(2.20 ± 0.35)	0.28	NS
	*Block2_ht3*	.	.	335(2.14 ± 0.07)	72(2.54 ± 0.19)	3(2.28 ± 0.82)	0.21	NS

Genotype and haplotype distributions and *P*-values controlling for sire and age at slaughter as covariates are shown.

C/C, C/R, and R/R represent the common allele, and heterozygotes and homozygotes for the rare allele, respectively.

N(LSMEAN ± SE): Number of animals (least square mean of values ± standard errors).

*P*^*cor*. ^represents the simple corrected *P*-value (Bonferroni correction).

Significant associations are shown in boldface. NS: not significant

Associations of *CAPN1 *polymorphisms with cold carcass weight (CW) and marbling score (MS) were analyzed using the mixed-effect model. The obtained *P-*values were corrected for multiple testing using the Bonferroni correction method. Two common haplotypes (*Block2_ht2 *and *Block2_ht3*) were also tested with the same covariates in a similar manner (Table 2). *Block1_ht1*, *Block1_ht2*, *Block1_ht3*, *Block2_ht1*, *Block2_ht4*, and *Block2_ht5 *were not analyzed because they were equivalent with *c.630A>G (T210T)*, *c.579G>A (K193K)*, *c.843+330A>G*, *c.760-24T>C*, *c.1869+235C>G*, c.2151*832G>A, and c.2151*479C>T, respectively (Figure 1B).

No positive associations were detected with CW. However, one polymorphism (c.2151*479C>T) in 3'UTR showed significant associations with MS. The "T" allele of c.2151*479C>T revealed an additive effect on MS, i.e., the lowest MS was found in "T/T" (MS = 0.94), intermediate in "C/T" (MS = 1.56), and the highest in "C/C" (MS = 2.34) (*P*^*cor*. ^= 0.02). In further haplotype association analysis, no positive associations were detected with CW and MS (Table 2).

Four polymorphisms, G316A, V530I, C4685T and C4751T have shown significant effects on meat tenderness [10,12-16]. However, one potential SNP marker with a reported effect on meat tenderness, specifically G316A in exon 9 of the CAPN1 gene, which produces a substitution of alanine for glycine at position 316 of the CAPN1 protein, was homozygous in our population (data not shown). In addition, V530I, C4685T and C4751T were discovered in Korean cattle (*c.1588G>A *[V530I], *c.1611+104C>T *and *c.1800+169C>T *respectively), and C4685T and C4751T are in absolute LD with each other in this study (Figure 1A). But these SNPs showed no association with MS. Interestingly, the results of our study showed that one polymorphism (c.2151*479C>T) in 3'UTR was associated with MS.

Although the mechanisms involved in the association of polymorphisms in the UTR region with MS are not currently understood, our study clearly indicates that c.2151*479C>T has an influence on MS in Korean cattle in light of the fact that strong association (*P*^*cor*. ^= 0.02), high enough to overcome the correction for multiple testing, was detected. We can hypothesize that polymorphisms in the UTR region might impact gene function by affecting the mRNA stability as well as regulatory motifs within UTRs. In addition, although it is true that the allele itself may be functional and directly affect the expression of the phenotype, a more likely event is that the allele is in linkage disequilibrium with another allele at a nearby locus that is the true causal allele.

Our results may be a false positive test because there is no quantitative trait locus (QTL) evidence for marbling in the respective genome region and *CAPN1 *is not a convincing functional candidate gene for marbling. However, similar to other association studies using the candidate gene strategies, although not complete, would facilitate further studies and contribute to understanding the genetic background of important traits. Further biological and/or functional evidence is needed to confirm the genetic effects of *CAPN1 *polymorphisms on MS.

## Conclusion

In summary, we have identified 39 polymorphisms in *CAPN1 *in Korean cattle, and 12 common polymorphic sites were selected for genotyping. Statistical analysis revealed that one polymorphism (c.2151*479C>T) in 3'UTR showed significant associations with the important carcass quality trait, marbling score, in Korean cattle.

## Methods

### Animals and phenotypic data

The Korean native cattle genomic DNA samples were obtained from 421 steers produced from 76 sires used in the progeny testing program of the National Livestock Research Institute (NLRI) of Korea. All steers were fed for 731.39 ± 16.53 days under a tightly controlled feeding program in the Daekwanryeong and Namwon branches. The average birth date was May 10 [range: April 15 – June 3]. After two years, animals were slaughtered in May 3 to 18. They were weaned at a mean age of 3 months and fed until they were 6 months old with 30% concentrates and 70% roughage. After 6 months of age, they were fed with concentrates consisting of 15% crude protein (CP)/71% totally digestible nutrients (TDN) until they were 14 months old; 13% CP/72% TDN until 20 months; and 11% CP/73% TDN until 24 months of age. The roughage was offered *ad libitum*, and steers had free access to fresh water during the whole period. Live weights were determined before slaughter. The mean of the live weights was 539.93 ± 51.96 kg. Yield grades for carcasses were determined by cold carcass weight (CW). After a 24-h chill, CW was measured, and then the left side of each carcass was cut between the last rib and the first lumbar vertebra to determine marbling score (MS). The mean of the CW was 311.47 ± 33.39 kg. MS was determined by assessing the degree of marbling in the cut surface of the ribeye. The degree of marbling was evaluated according to the Korean Beef Marbling Standard (1 = trace, 7 = very abundant) [17]. The mean of the MS was 2.25 ± 1.36.

### Sequencing analysis of *CAPN1*

We have sequenced exons and their flanking regions to discover variants in 24 unrelated Korean native cattle using the ABI PRISM 3730 DNA analyzer (Applied Biosystems, Foster City, CA). Sixteen primer sets for the amplification and sequencing analysis were designed based on GenBank sequences (Ref. Genome seq. NC_007330 released on 26 Dec. 2006 and Ref. mRNA seq. AF221129 released on 9 Feb. 2000). Primer information is given in supplementary data. Sequence variants were verified by chromatograms [see Additional file 1].

### Genotyping by single-base extension (SBE) and electrophoresis

For genotyping of polymorphic sites, amplifying and extension primers were designed for single-base extension (SBE) [18]. Primer extension reactions were performed with the SNaPshot ddNTP Primer Extension Kit (Applied Biosystems). To stop the primer extension reaction, one unit of SAP (shrimp alkaline phosphatase) was added to the reaction mixture, and the mixture was incubated at 37°C for 1 hour, followed by 15 min at 72°C for enzyme inactivation. The DNA samples containing extension products and GeneScan 120 Liz size standard solution were added to Hi-Di formamide (Applied Biosystems) according to the recommendation of the manufacturer. The mixture was incubated at 95°C for 5 min, followed by 5 min on ice, and then electrophoresis was performed using the ABI Prism 3100 Genetic Analyzer. The results were analyzed using the programs ABI Prism GeneScan and Genotyper (Applied Biosystems). Probe information is given in supplementary data [see Additional file 1].

### Statistics

The χ^2 ^tests were used to determine whether the individual variant was in equilibrium at each locus in the population (Hardy-Weinberg equilibrium). We examined a widely used measure of linkage disequilibrium between all pairs of biallelic loci, D' (the correlation coefficient [Delta, |D'|]), LOD (logarithm of odds), and r2. Strength of LD between pairs of SNPs was measured as D' using Haploview. Regions of strongly associated markers (LD blocks) were inferred by Gabriel's method as implemented in Haploview [19,20]. Gabriel's method defines pairs of SNPs to be in strong LD if the one-sided 95% D' confidence bound is between 0.7 and 0.98. The method defines a block if 95% of pairwise SNP comparisons are in strong LD. r2 was also used to determine whether the pairs of sites were in absolute LD. Haplotypes and their frequencies were inferred using the algorithm developed by Stephens et al. [21]. Phase probabilities of each site were calculated for each individual using this software (PHASE) (input option: ignoring families). Phase probabilities of all polymorphic sites for haplotypes were calculated for each individual using this software. Because 95% of samples had phase probabilities greater than 97%, 97% was chosen as threshold for phase probability. Associations between individual SNPs and carcass traits (CW and MS) were determined by the mixed effect model, treating "sire" as a random effect, and "age" at slaughter was also included in the model as a covariate using the library (nlme) in the R statistical package (see Availability and requirements for URL). Other covariates were not available for this analysis. We used a single SNP model. Single SNP/haplotype effects were tested in the mixed effect model. P-values were corrected by Bonferroni correction for multiple testing (the threshold of raw P-value significance was 0.0018 [14 polymorphisms and haplotypes, and 2 phenotypes analyzed]). A Type I error of 5% was used to obtain the Bonferroni corrected p-value. For the haplotype analyses, we fit the model with the same covariates in a similar manner.

## Availability and requirements

R statistical package: 

## Authors' contributions

HSC carried out the statistical analyses and drafted the manuscript. LHK, SN, HWL, CSH, and JOK carried out the sequencing, SNP discovery, and genotyping. D-HY and I-CC participated in the animal and phenotypic data collection. HDS participated in the design and coordination, and helped to draft the manuscript. BLP and JSB carried out further statistical analyses. All authors read and approved the final manuscript.

## Supplementary Material

Additional file 1 Supplementary information to CAPN1. The data provided represent primer information for CAPN1 sequencing, genotyping probe and chromatograms of each SNP. Click here for file
