# Supplementary material for: A single nucleotide polymorphism in CAPN1 associated with marbling score in Korean cattle
Source: BMC Genet. 2008 Apr 19;9:33. doi: 10.1186/1471-2156-9-33 (PMC2386817; doi:10.1186/1471-2156-9-33)

Ad. Table 1. Primer sequences for *CAPN1* sequence variants screening

| Primer | Position | Sequence | |
| --- | --- | --- | --- |
| CAPN1_1P | Exon1 | Forward | CGGGGACTACTCTGCCTTTG |
|  |  | Reverse | TCCCCAGACTAGCCAAGCTC |
| CAPN1_2P | Exon2 | Forward | GTGAAGAGGTGGGGAAACCA |
|  |  | Reverse | GAACGGGCACAGGGAACTAC |
| CAPN1_3P | Exon3-4 | Forward | GGGTCTCTTGCAGGAGCTGT |
|  |  | Reverse | GCTGAAATACCTGCCCAACC |
| CAPN1_4P | Exon5-6 | Forward | GAGGGATCTCAAAGCAGGGA |
|  |  | Reverse | CATGGCCTCAGATGACAGGA |
| CAPN1_5P | Exon7 | Forward | TCATTGGTGGAGTAGCCAGC |
|  |  | Reverse | GTCACTCCAGGCTCCTGTCC |
| CAPN1_6P | Exon8 | Forward | TATCTCCCAGGGACTCTCGC |
|  |  | Reverse | AGGAGCTGCAGGAAAAGGG |
| CAPN1_7P | Exon9-10 | Forward | TAGAGGCTGGGCAGGTCAGT |
|  |  | Reverse | CACACTTTTTCATCCTCAAAGCA |
| CAPN1_8P | Exon11 | Forward | CAGCTCCAGCTTGCTCTCTT |
|  |  | Reverse | TGTGTACCTCAGGGGTGTGA |
| CAPN1_9P | Exon12 | Forward | ACAGGTCTGAGGCAACAGCA |
|  |  | Reverse | CGCAAAGAGTCGAACGTGAC |
| CAPN1_10P | Exon13-14 | Forward | GAGGTCTGGGTCTGGGTCTG |
|  |  | Reverse | TTGTTATGGAGGAAACCGGG |
| CAPN1_11P | Exon15-16 | Forward | ATTTACCCAAGGTCACGCAG |
|  |  | Reverse | GGTGGGAGAAGCCTAGATCC |
| CAPN1_12P | Exon17 | Forward | CCCTTCCCTGCATGTTTCTA |
|  |  | Reverse | GAGGGGTGTTCTCTGAGTGC |
| CAPN1_13P | Exon18-19 | Forward | CAGTACTCACTCACCCAGCG |
|  |  | Reverse | ACTTCACCACCACTGCCATC |
| CAPN1_14P | Exon20-21 | Forward | GGTCGTTCTGGGTCCTCAAG |
|  |  | Reverse | TGCTGTGTCTGTGCAGGATG |
| CAPN1_15P | Exon22 | Forward | CCCTTGACAGAGGGAGGAGA |
|  |  | Reverse | CAGGCTAGGCTGCTTCTGGT |
| CAPN1_16P | Exon22 | Forward | CCACATCCTGACGTGTCCTC |
|  |  | Reverse | GAATGTGCAGGGAAGGAAGG |

Ad. Table 2. Sequences of amplifying and extension primers for genotyping of polymorphisms in *CAPN1* by single-base extension method

| Locus | Sequence | |
| --- | --- | --- |
| *c.579G>A (K193K)* | Forward | GAGGGATCTCAAAGCAGGGA |
|  | Reverse | CATGGCCTCAGATGACAGGA |
|  | Extension | GATGATGAGTTCTGGAGCGCCCTGCTGGAGAA |
| *c.630A>G (T210T)* | Forward | GAGGGATCTCAAAGCAGGGA |
|  | Reverse | CATGGCCTCAGATGACAGGA |
|  | Extension | CGAGGCCCTCTCAGGAGGCAGCAC |
| *c.760-24T>C* | Forward | TCATTGGTGGAGTAGCCAGC |
|  | Reverse | GTCACTCCAGGCTCCTGTCC |
|  | Extension | TGGGGGTGGGGACAGCGGC |
| *c.843+330A>G* | Forward | TCATTGGTGGAGTAGCCAGC |
|  | Reverse | GTCACTCCAGGCTCCTGTCC |
|  | Extension | TAATCAATGATGATCCTGGTAGTTCACCTGTTCCCCAGAGCTCA |
| *c.1199G>A (R400Q)* | Forward | CAGCTCCAGCTTGCTCTCTT |
|  | Reverse | TGTGTACCTCAGGGGTGTGA |
|  | Extension | CAATGATGATTTCTGGGTGAACCCCCAGTTCAAGATCC |
| *c.1588G>A (V530I)* | Forward | GAGGTCTGGGTCTGGGTCTG |
|  | Reverse | TTGTTATGGAGGAAACCGGG |
|  | Extension | TTATAATCAATGATGATACCCTCTGCAGAGAGCTGGATGACCAG |
| *c.1611+104C>T* | Forward | GAGGTCTGGGTCTGGGTCTG |
|  | Reverse | TTGTTATGGAGGAAACCGGG |
|  | Extension | CTCCTGGCAGGAGCCATACA |
| *c.1869+235G>C* | Forward | CAGTACTCACTCACCCAGCG |
|  | Reverse | ACTTCACCACCACTGCCATC |
|  | Extension | ATGATGATGCCGAGCCCTCACCCTCTGCCCCC |
| *c.2151*479C>T* | Forward | CCACATCCTGACGTGTCCTC |
|  | Reverse | GAATGTGCAGGGAAGGAAGG |
|  | Extension | TGCCTGTGCGGAAGCCAATG |
| *c.2151*765A>G* | Forward | CCACATCCTGACGTGTCCTC |
|  | Reverse | GAATGTGCAGGGAAGGAAGG |
|  | Extension | GTTTCTGCCTAAACCTGCTGGCCCAC |
| *c.2151*832G>A* | Forward | CCACATCCTGACGTGTCCTC |
|  | Reverse | GAATGTGCAGGGAAGGAAGG |
|  | Extension | TCTCCTTCCCCATCGCCCCAGTGGCT |
| *c.2151*845A>G* | Forward | CCACATCCTGACGTGTCCTC |
|  | Reverse | GAATGTGCAGGGAAGGAAGG |
|  | Extension | AGTGGCTGGCACCTCTGCCC |

Ad. Figure 1. Chromatograms of discovered polymorphisms in *CAPN1*


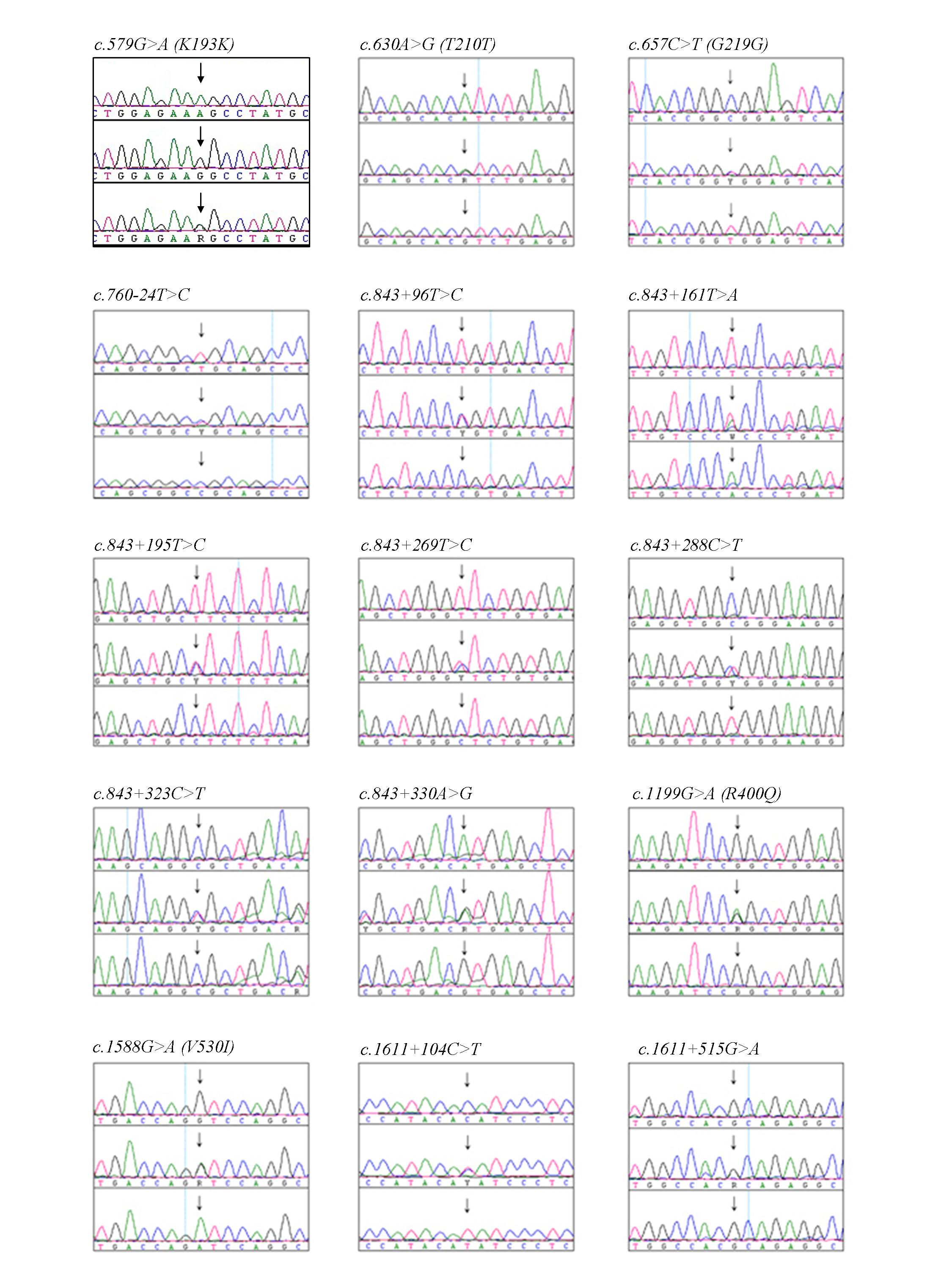


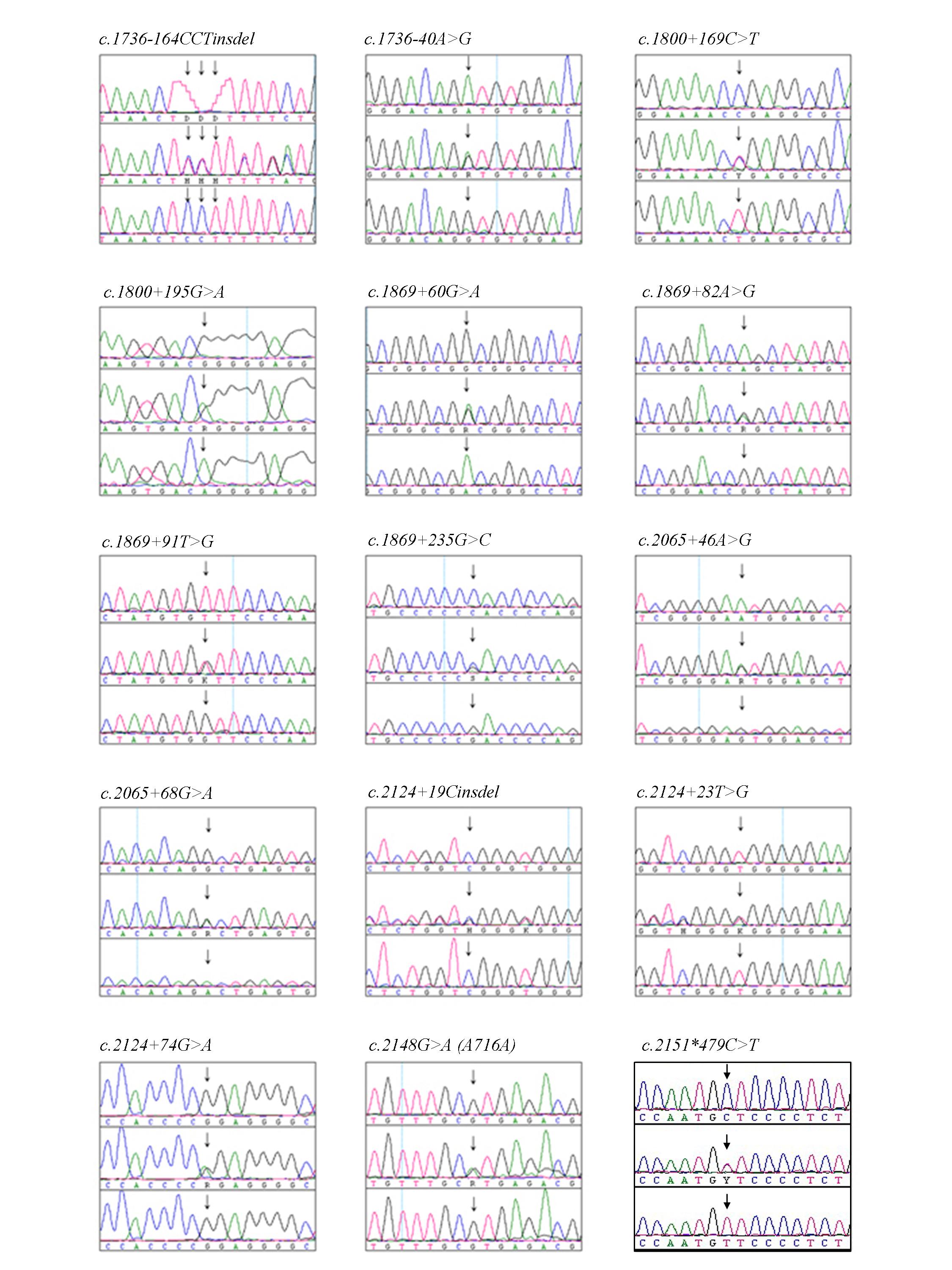


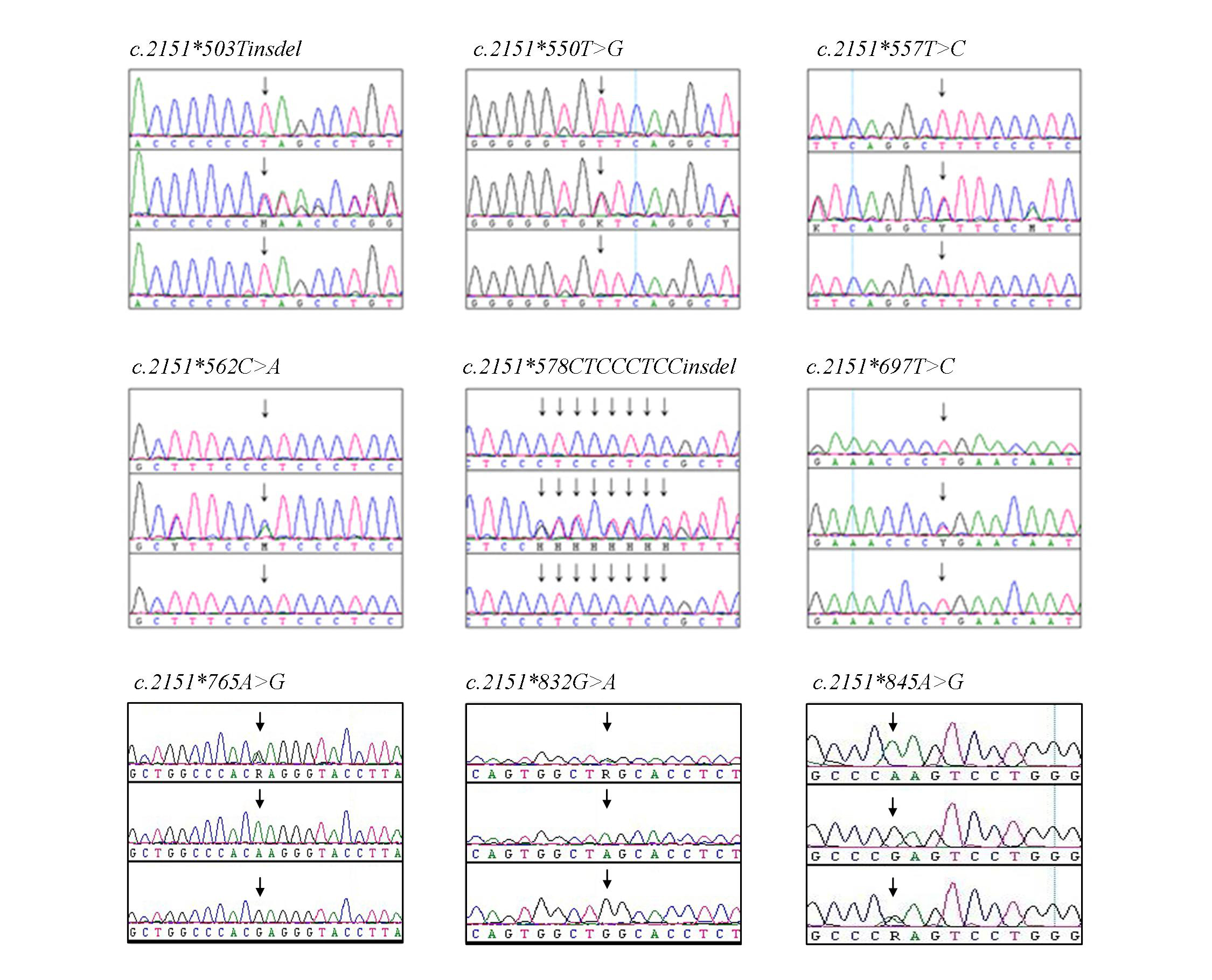

Supplement: Additional file 1 — Supplementary information to CAPN1. The data provided represent primer information for CAPN1 sequencing, genotyping probe and chromatograms of each SNP. [file 1471-2156-9-33-S1.doc]
